# Response to Anastrozole Treatment in a Case with Peutz-Jeghers Syndrome and a Large Cell Calcifying Sertoli Cell Tumor

**DOI:** 10.4274/jcrpe.3625

**Published:** 2017-06-01

**Authors:** Merve Koç Yekedüz, Zeynep Şıklar, Berk Burgu, Zarife Kuloğlu, Pınar Kocaay, Emine Çamtosun, Mehmet İsakoca, Aydan Kansu, Tarkan Soygür, Merih Berberoğlu

**Affiliations:** 1 Ankara University Faculty of Medicine, Department of Pediatrics, Ankara, Turkey; 2 Ankara University Faculty of Medicine, Department of Pediatric Endocrinology, Ankara, Turkey; 3 Ankara University Faculty of Medicine, Department of Pediatric Urology, Ankara, Turkey; 4 Ankara University Faculty of Medicine, Department of Pediatric Gastroenterology, Ankara, Turkey

**Keywords:** Peutz-Jeghers syndrome, large-cell calcifying Sertoli cell tumor, prepubertal gynecomastia, anastrozole

## Abstract

Peutz-Jeghers syndrome (PJS) is inherited as an autosomal dominant trait characterized by multiple gastrointestinal hamartomatous polyps, mucocutaneous pigmentation, and an increased risk of neoplasm. Large-cell calcifying Sertoli cell tumor (LCCSCT) is a kind of sex cord-stromal tumor which may co-exist with PJS and which is characterized radiologically by calcification foci within the testes. Surgical treatment options for this tumor range from testis-preserving surgery to radical orchiectomy. Not with standing this invasive approach, recently, there are some case reports demonstrating the efficacy of aromatase inhibitors in avoiding orchiectomy and its associated complications. In this paper, we have presented a LCCSCT case diagnosed in a boy with PJS and his response to anastrozole treatment.

## What is already known on this topic?

Large-cell calcifying Sertoli cell tumor (LCCSCT) is a kind of sex cord-stromal tumor and can be encountered in Peutz-Jeghers syndrome (PJS) patients.

## What this study adds?

Without diagnostic biopsy, gynecomastia due to LCCSCT in PJS can be treated successfully with anastrozole treatment.

## INTRODUCTION

Peutz-Jeghers syndrome (PJS), inherited as an autosomal dominant trait, is characterized by multiple gastrointestinal hamartomatous polyps, mucocutaneous pigmentation, and an increased risk of neoplasm (1,2).

PJS may be accompanied by endocrine conditions such as precocious puberty, gynecomastia, adrenocortical hyperplasia, and pituitary adenoma. Gynecomastia may refer to a clinical manifestation of a large-cell calcifying Sertoli cell tumor (LCCSCT) in PJS patients (2).

The exact mechanism that underlies the development of the LCCSCT remains unknown. LCCSCT accounts for 0.4-1.5% of all testicular tumors (3). Oftentimes, it appears in a bilateral and multifocal manner prior to, in the course of, or in the late phase of puberty. LCCSCT is radiologically characterized as a sex cord-stromal tumor by the calcification foci within the testes.

The polymorphism in the gene encoding a key enzyme in the estrogen biosynthesis, namely CYP 19 (P450 19), stimulates the increased levels of estrogen and is held responsible for the clinical findings associated with PJS. In approximately 60% of patients, gene mutation is identified in the LKB1/STK 11 tumor suppressor gene. Other genes, undefined for the time being, may also be responsible for the pathogenesis (3,4,5,6).

LCCSCTs develop in a minority of PJS cases. The first finding of PJS, on the other hand, might be related to LCCSCT. Under physiological conditions, Leydig cells represent the source of aromatase enzyme production in the testes, while in LCCSCT patients, they are expressed particularly by neoplastic Sertoli cells. Elevated expression of aromatase enzyme clinically causes gynecomastia in males. The estrogen level, however, is not necessarily found to be increased (7).

The surgical treatment options for this tumor vary in a range of techniques from testis-preserving surgery to radical orchiectomy. However, today, medical treatment is opted as the first choice. Treatment with aromatase inhibitors (anastrozole) is among the options (1,2,3,7,8,9). Anastrozole treatment is effective in controlling LCCSCT (1). Aromatase inhibitors may help in avoiding orchiectomy and its associated complications. There is a limited number of cases reported in the literature (1,2,3,7,8,9).

## CASE REPORT

A 9.5-year-old male patient was brought to our department with a complaint of enlargement of the breast tissue noted in the past 6 months. The history of the patient revealed that a circumoral pigmentation had been detected at the age of 2.5 years and that the patient was referred to a department of pediatric gastroenterology when he was 4 years old at which time he was diagnosed to have PSJ upon findings during endoscopic investigation which revealed presence of three rectal hamartomatous polyps and ultrasonographic (USG) findings which showed multiple paraaortic, mesenteric, hypoechoic lymph nodes.

Results of body measurements revealed a height standard deviation score (SDS) of 0.34 and a body mass index (BMI) of 82.48%. Hyperpigmented lesions were noted around the mouth, on the lower lip, and on the buccal mucosa. On pubertal examination, testicular volumes were 3 mL and 3 mL, and pubic hair was at stage P1. The patient had breast growth, with a diameter of 2 cm on both sides, in compliance with bilateral gynecomastia. Bone age was assessed as 9 years.

Laboratory testing resulted as follows: Luteinizing hormone (LH): 0.31 mIU/L (N: <0.3 for prepubertal), follicle-stimulating hormone (FSH): 0.4 mIU/L (N: 0.21-4.33), estradiol <20 pg/mL (N: <20 pg/mL) (sensitivity for ADVIA^®^ is between 11.8 to 3000 pg/mL), estrone <0.04 nmol/L (N: 0.03-0.22), beta human chorionic gonadotropin: 0.11 mIU/L (N: <5), total testosterone <10 ng/dL (N: <20 for prepubertal), 17-hydroxylase progesterone: 0.6 ng/mL (N: ≤90), dehydroepiandrosterone sulfate: 55.5 mg/dL (N: ≤91), AFP: 1.57 ng/mL (N: <6), inhibin B: 200 pg/mL (N: 35-170), inhibin α: 0.4 pg/mL (N: <2), and prolactin: 18.53 ng/mL (N: <20 ng/mL). No hormonal pathology was detected to explain the gynecomastia. Unfortunately, we did not have the means to investigate presence of polymorphism in the *CYP19* gene and mutations in the LKB1/STK 11 tumor suppressor gene.

According to scrotal USG imaging results, the size and dimensions of the right testis were 22.4x14.1x9.8 mm (1.6 mL) and those of the left testis 21.9x13.3x11.2 mm (1.5 mL). In addition, coarse calcification of a size of 1.4-1.8 mm in compliance with microlithiasis and a relatively well-circumscribed hypoechoic nodular formation of 3 mm at the right testis were noted. No typical vascularity implying a neoplasia was detected.

As a result of our assessment in collaboration with the department of pediatric urology, based on the past diagnosis of PJS, detection of gynecomastia, bilateral coarse calcification of the testes as imaged by scrotal USG, the patient was considered most likely to have a LCCSCT. The department of pediatric urology did not recommend a biopsy to be performed taking into account the technical difficulty arising from the small size of the lesion and the potential risk of alteration of lymphatic drainage due to transscrotal biopsy which could also lead to testicular damage and eventually orchiectomy. Joint decision was established to initiate treatment with anastrozole (1 mg/day). After six months of treatment, gynecomastia was observed to have remarkably regressed. After one year of treatment (calendar age: 10.8 years), the height SDS of the patient was 0.11 and his BMI was 82%. On pubertal examination, bilateral testicular volumes were 4 mL, penile length was measured to be 6 cm, and pubic hair appearance was at pubarche stage P1. On the left side, gynecomastia had totally regressed, while its size was reduced to 0.5 cm on the right side. Throughout the follow-up, the patient’s somatic development, height and weight curves remained within the age-matched interval with a parallel course to the percentile line. Laboratory testing resulted as follows: LH: <0.2 mIU/L, FSH: <0.2 mIU/L, total testosterone: <10 ng/dL, E2:<20 pg/mL, and was evaluated to be normal. No change was viewed on scrotal USG imaging of the solid lesion in the right testis. After one year of treatment, it was decided to discontinue anastrozole treatment, since the clinical findings were found to be improved.

No gynecomastia increase occurred during the 6-month follow-up after treatment discontinuation. During the 1.5 years of follow-up without treatment, the growth of the patient showed a trend in accordance with his age.

## DISCUSSION

In males with PJS, presence of Sertoli cell tumor may manifest as gynecomastia and feminization-related findings (1,2,7). The first PJS case with Sertoli cell tumor-associated feminization findings was described by Cantú et al in 1980 (8). Until today, 30 comparable cases have been reported (1,3,9). In PJS patients, the functioning of Sertoli cells varies, consequently leading to clinical variance. While gynecomastia may appear as the first finding of the Sertoli cell tumors, it may not appear at all, despite the existence of the tumor.

Testicular tumors are quite rare in the prepubertal period. In patients with PJS, however, endocrinological findings such as prepubertal gynecomastia might be indicative of a testicular tumor, namely, LCCSCT. Malignancy is found in approximately 17% of patients with LCCSCT but is rare in young patients with bilateral tumors or in association with a genetic syndrome including PJS (10). Despite the low risk of malignancy in our patient, we planned biannual USG examination of the testes in addition to clinical and laboratory follow-up.

The polymorphism in the gene encoding a key enzyme in the estrogen biosynthesis, namely CYP 19 (P450 19), stimulates the increase in estrogen levels and is held responsible for the clinical findings associated with PJS. Elevated aromatase activity not only causes gynecomastia but also leads to other undesired effects such as increased rates of linear growth and bone maturation (1,2,3,7,8,9).

Local estrogen biosynthesis within the breast can be highly variable. It has been suggested that small amounts of estrogen may be sufficient to induce breast enlargement. In addition, increased bioavailability, local biosynthesis, or tissue responsiveness might be the factors responsible for normal estrogen levels despite the probable elevated aromatase activity (9).

In our patient, a prior diagnosis of PJS, presence of prepubertal gynecomastia of recent onset, and appearance of bilateral coarse calcifications in scrotal USG were the factors suggestive of a diagnosis of LCCSCT.

When the department of pediatric urology was consulted, we have been notified that despite a suspected malignancy, implementation of a biopsy procedure might result in an altered lymphatic drainage and lead to testicular damage and complications which may ultimately require orchiectomy. For the purpose of avoiding such complications, anastrozole treatment was initiated for LCCSCT without performing a biopsy. Although biopsy is an intervention which allows establishment of a definite diagnosis (4,11), LCCSCT diagnosis based on clinical and laboratory findings should be kept in mind especially for patients with PJS.

Anastrozole treatment was reported to be effective in achieving control over clinical findings of LCCSCT cases with gynecomastia (2,9,12). Treatment was initiated for our patient. After six months of treatment with anastrozole (1 mg/day), gynecomastia was observed to have remarkably regressed. No gynecomastia increase occurred during the 6-month follow-up after treatment discontinuation.

PJS was diagnosed in a 7.5-year-old patient showing painless gynecomastia when buccal pigmentation was noticed (2). In this patient, multiple parenchymal calcifications were noted in scrotal USG. Unlike our case, estradiol level was high in that patient. Testicular biopsy results were in compliance with a Sertoli cell tumor and aromatase treatment was initiated. After one year of treatment, initially with testolactone, followed by anastrozole, the breast diameter of the patient decreased from 7 cm to 3 cm, and, in parallel, testicular volume receded from 5 mL to < 4 mL. Although elevated estradiol levels may be helpful in the diagnosis, these levels are not necessarily increased in all LCCSCT cases, as was the case in our patient (9).

In cases with PJS who develop LCCSCT, elevated aromatase activity not only causes gynecomastia but also leads to other undesired effects such as increased rates of linear growth and bone maturation (1,2,3,7,8,9). After 2 years of anastrozole treatment, 9-year-old twins with PJS who were diagnosed to have LCCSCT upon occurrence of prepubertal gynecomastia, were found to have a decrease in rate of growth (1,13). Somatic growth of our case was followed-up due to the risk of potential growth retardation; he was found to have normal height and weight and his growth curve followed a normal course. Also in our patient, no increase was noted in gynecomastia after 6 months without treatment. However, new studies are needed to assess the probability of gynecomastia relapse beyond the cessation of treatment. No side effect was reported following anastrozole treatment in a dose of 1mg/day for two years (1). Long-term effects constitute another subject field which needs to be investigated. Anastrozole treatment is more commonly administered in adult breast cancer patients. There are a plethora of studies in the above-mentioned field, but the number of studies regarding its adverse effects in patients with LCCSCT and prepubertal gynecomastia is limited (1,2,3,7,8,9,13,14). Therefore, considering the reported side effects of these drugs, follow-up of growth and of bone density should be conducted in patients treated with aromatase inhibitors.

In conclusion, LCCSCT diagnosis based on the clinical and laboratory findings is feasible. This is particularly important for patients who are susceptible to the risks of a biopsy procedure. Aromatase inhibitors are effective in treatment of gynecomastia induced by LCCSCT accompanying PJS. Anastozole treatment lasting for one year does not have a negative impact on growth. Medical treatment may help in avoiding orchiectomy and its associated complications. However, there is need for longer follow-up studies and more extensive information regarding the efficacy of medical treatment and its long-term effects.
